# A DASA displaying highly efficient and rapid reversible isomerization within sustainable nano/micro capsules: one step closer to sustainability†

**DOI:** 10.1039/d4sc04868g

**Published:** 2024-09-25

**Authors:** Baoshuo Liu, Xinnian Fan, Hao Ma, Yutong Xie, Haojun Fan, Qiang Yan, Jun Xiang

**Affiliations:** a College of Biomass Science and Engineering, Sichuan University Chengdu 610065 China junxiang@scu.edu.cn; b State Key Laboratory of Molecular Engineering of Polymers, Department of Macromolecular Science, Fudan University Shanghai 200433 China; c State Key Laboratory of Polymer Materials Engineering, College of Polymer Science and Engineering, Sichuan University Chengdu 610065 China; d High-Tech Organic Fibers Key Laboratory of Sichuan Province Chengdu 610041 China

## Abstract

Donor–acceptor Stenhouse adducts (DASAs), derived from bio-based furfural, demonstrate reversible isomerization when exposed to light and heat, positioning them as attractive candidates for sustainable smart materials. However, achieving efficient and rapid isomerization in high bio-content solid-state matrices, especially under mild conditions, remains a significant hurdle due to restricted molecular mobility and limited matrix options. To address this, we developed a novel solid matrix in the form of sustainable nano/micro capsules, which boast the highest bio-content reported to date (57%). Composed of polymethylmethacrylate (PMMA) and a lauric–stearic acid eutectic mixture (L–SEM), these capsules facilitate highly efficient and rapid reversible isomerization of a third-generation DASA (DASA-1). Remarkably, the system achieves 84% forward and 90% reverse isomerization under mild temperatures, significantly enhancing the material's photo-switching capabilities. This advancement not only addresses the critical challenge of isomerization within high bio-content solid matrices but also opens broader possibilities for the application of bio-based DASAs in environmentally friendly technologies, such as color-rich rewritable papers. By innovating in the design of sustainable smart materials, this work has the potential to extend the utility of DASAs across various scientific fields, contributing to the global shift towards a low-carbon, environmentally sustainable society.

## Abstract 2

A donor–acceptor Stenhouse adduct exhibits highly efficient, rapid, and reversible isomerization within sustainable nano/micro capsules. Forward isomerization reaches 84% (620 nm, 25 °C, 300 s), and backward reaches 90% (50 °C, 120 s).

## Graphical abstract

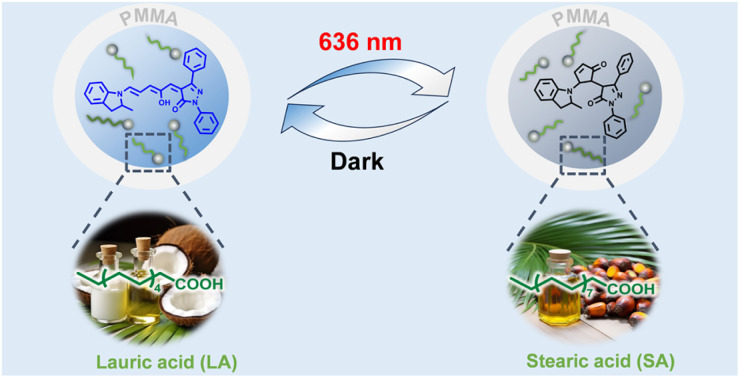

## 1. Introduction

The development of smart materials capable of dynamically responding to external stimuli represents a frontier in materials science, offering transformative applications across various domains.^1–4^ Among these materials, Donor–Acceptor Stenhouse Adducts (DASAs) have gained significant attention since their discovery in 2014.^5,6^ Their ability to respond to specific wavelengths of light and thermal stimuli, combined with negative photochromism, highly tunable performance, and derived from bio-based furfural, has sparked growing research aimed at controlling their isomerization behavior and expanding their potential applications.^7–17^ As a result, DASAs are now recognized as leading candidates for the development of sustainable smart materials.

A critical factor for the successful development of DASA-based smart materials lies in their performance within solid matrices—a topic that has gained increasing attention in recent years. The primary challenge in this area is that solid matrices often restrict molecular mobility and introduce intermolecular interactions, both of which can severely limit their ability to undergo unrestricted isomerization.^5^ To overcome these limitations, innovative matrix engineering strategies have become essential, enabling researchers to unlock the full potential of DASAs for advanced applications.

Current research on solid matrices for DASAs has largely focused on two categories: amorphous polymers and porous crystals.^16,18–33^ Amorphous polymers, particularly those with low glass transition temperatures,^22^ have proven effective in enhancing molecular mobility, thereby facilitating efficient isomerization. In contrast, crystalline matrices have received relatively less attention. A few examples, such as metal– and covalent–organic frameworks (MOFs/COFs) with low-polarity porous environments, have demonstrated varying degrees of success in enabling the reversible isomerization of DASAs.^30–33^ A critical but often overlooked factor in the development of DASAs is the bio-content of the matrices that host them. While DASAs themselves leverage the bio-based platform molecule “furfural”, which can be sourced from agricultural waste like corn cobs or rice husks,^34–36^ the matrices typically rely on non-renewable resources. As shown in Table S1,† this reliance contradicts the global push for low-carbon development, which advocates for the use of renewable biomass over petroleum-based materials.^37–40^ To address this issue, our previous work introduced a plant oil-based polymer matrix with a bio-content of 52%,^20^ enabling the highly efficient and rapid reversible isomerization of a second-generation DASA. Building on this initial success, this work aims to further increase the bio-content of solid matrices, advancing the development of more sustainable DASA-based materials that align with both environmental goals and the future of advanced materials science.

Beyond the bio-content, a critical factor for success is whether the solid matrices can support efficient isomerization of DASAs. Despite the progress made, it remains challenging to achieve high efficiency (>80%) and rapid reversible isomerization (time to reach the efficiency ≤300 s) of DASAs within solid matrices under mild conditions (<100 °C). This limitation has become a significant hurdle in advancing the development of sustainable DASA-based smart materials.

**Fig. 1:**
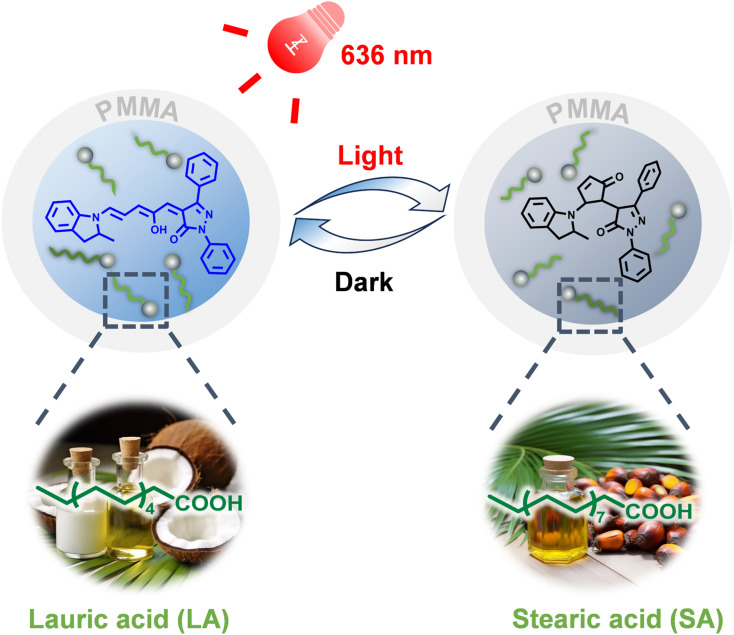
Schematic illustration showing the highly efficient and rapid reversible isomerization of DASA-1 within sustainable nano/micro capsules of PMMA/L–SEM.

To overcome this challenge, we present a novel approach that surpasses traditional solid matrices by utilizing a eutectic mixture of lauric–stearic acid (L–S, 4 : 1, w/w, m.p., ∼40 °C), which is sustainably sourced from natural coconut and palm oils. Encapsulated within nano/micro polymethylmethacrylate (PMMA) capsules, this composite matrix features the highest bio-content reported to date (57%), providing a unique combination of enhanced molecular mobility and structural stability through polymer encapsulation. As demonstrated in Fig. 1, a third-generation DASA, termed DASA-1, exhibits highly efficient and rapid reversible isomerization within capsules under 636 nm irradiation and mild heating (50 °C). A significant finding is that the inclusion of PMMA not only enhances the thermal stability of the DASA-1-doped eutectic mixture at elevated temperatures but also improves isomerization efficiency. Furthermore, we successfully leveraged this encapsulation technique to develop a smart material: a color-rich rewritable paper. This innovation has the potential to address the environmental impact of traditional paper production, which consumes significant tree resources and represents a promising step toward more sustainable and recyclable materials.

## 2. Results

### 2.1. DASA-1 displays extremely efficient reversible isomerization in dichloromethane

**Fig. 2:**
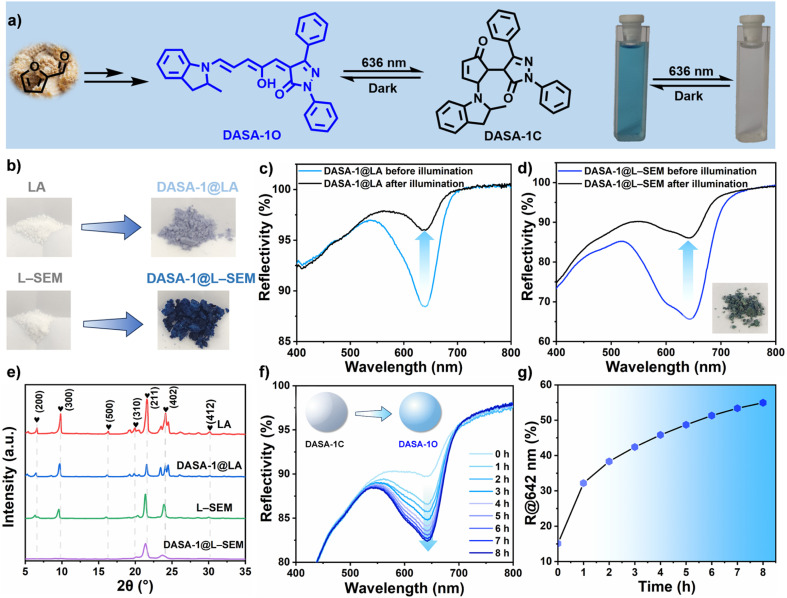
(a) DASA-1 exhibits extremely efficient reversible isomerization in DCM. (b) Photos of powdery LA and L–SEM before (left) and after (right) doping with DASA-1. Reflectance spectra of DASA-1@LA (c) and DASA-1@L–SEM (d) before and after 636 nm irradiation (41 mW cm^−2^, 5 min). The inset in (d) shows the sample of DASA-1@L–SEM after irradiation. (e) XRD patterns of LA, DASA-1@LA, L–SEM, and DASA-1@L–SEM. (f) Reflectance spectral changes of DASA-1@L–SEM in the dark at 20 °C. (g) Thermal recovery of DASA-1@L–SEM at 20 °C in the dark.

The detailed synthesis and characterization of DASA-1 are given in ESI (Fig. S1–S3†) and its reversible isomerization is first investigated in dichloromethane (DCM), where its absorption band is located at 640 nm (Fig. S4†), rendering the solution a deep cyan hue. It is found that this solution turns into colorless (Fig. 2a) upon 636 nm irradiation, indicating the efficient forward linear-cyclic isomerization of DASA-1, namely switching from the colored, ground state linear isomer (DASA-1L) to the colorless closed-ring isomer (DASA-1C). Under ambient conditions, the backward cyclic-linear isomerization occurs in the dark. As revealed in Fig. S4 and S5,† both the forward and backward isomerization of DASA-1 is extremely efficient in DCM, reaching 95% and 98%, respectively.

### 2.2. DASA-1 displays reversible isomerization in LA and L–SEM

Our initial attempt involves embedding DASA-1 at a concentration of 3 wt% into pure LA (m.p. = 43 °C) (see ESI† for experimental details). Through reflectance spectrum analysis, the reflection band of the resulting sample, termed DASA-1@LA (Fig. 2b), emerged at 640 nm, which is consistent with the *λ*_max_ value observed in DCM. Fig. 2c exhibits the forward isomerization of DASA-1 reaches 73% after 5 min of irradiation, with gradual recovery observed in darkness (data not shown). However, it is worth noting that the coloration of DASA-1@LA is quite faint (Fig. 2b), which is primarily attributed to an exceedingly low proportion of DASA-1L within LA. To address this issue, LA was replaced with lauric–stearic acid eutectic mixture (L–SEM) (m.p., 40.3 °C) instead of elevating DASA-1's doping levels, due to concerns that higher concentrations could adversely affect its isomerization.^41,42^ Surprisingly, as depicted in Fig. 2b, DASA-1@L–SEM displays a significantly deeper hue (deep cyan), suggestive of an elevated content of DASA-1L within L–SEM. This deepening of color could plausibly be attributed to the replacement of certain LA molecules with SA, which features longer alkyl chains, thus reducing the density of carboxylic groups and decreasing the overall polarity. Since the polarity of the DASA-1L is lower than that of DASA-1C, this results in the observed enhancement of color in L–SEM. Additionally, another potential reason is that the partial substitution of LA with SA enables the solid matrix of L–SEM to more effectively stabilize the multiple photoisomerization intermediates^43^ of DASA-1.

To acquire more information, reflectance spectra were collected. Compared to that of DASA-1@LA, Fig. 2d reveals a notable alteration in the reflectance spectrum of DASA-1@L–SEM, with the band shape resembling that observed within DCM, albeit with a minor redshift to 642 nm and a broadening of the peak. These changes may originate from the induced intermolecular interactions between DASA-1 molecules and fatty acids. To verify this, XRD tests were further conducted, and the results indicate that both LA and L–SEM maintain their crystalline state at room temperature (Fig. 2e), both before and after doping with DASA-1. Like the XRD curve of LA,^44^ that of DASA-1@LA shows no new diffraction peaks and the peak positions remain substantially unchanged, suggesting that the integration of DASA-1 does not affect the preferred crystalline structure of LA. However, we observed changes in the relative intensities of the peaks, a phenomenon consistent with previous studies of LA doped with other substances.^45–47^ This change may stem from the influence of DASA-1 on the crystallinity of LA. In contrast, the DASA-1@L–SEM sample exhibits a distinct difference: the crystalline peaks below 10° are absent. This absence implies that DASA-1 changes the preferred crystalline structure of L–SEM or the incorporation of DASA-1 imposes a certain degree of structural restriction on the L–SEM phases, likely due to a higher proportion of the larger molecular dimensions of DASA-1 molecules (DASA-1L) existing in the matrix.

Does the structural confinement exert an influence on the reversible isomerization of DASA-1? To investigate this, DASA-1@L–SEM was subjected to 636 nm irradiation. Mirroring its behavior in pure LA, the efficiency of the forward isomerization of DASA-1 reaches ≈71% (Fig. 2d). However, it takes 5.5 h to return to half of its initial value at 20 °C (Fig. 2f and g), indicating that structural confinement slows the backward isomerization rate of DASA-1.

### 2.3. Enhanced stability at elevated temperature was achieved by the encapsulation of DASA-1 and L–SEM into nano/micro capsules

**Fig. 3:**
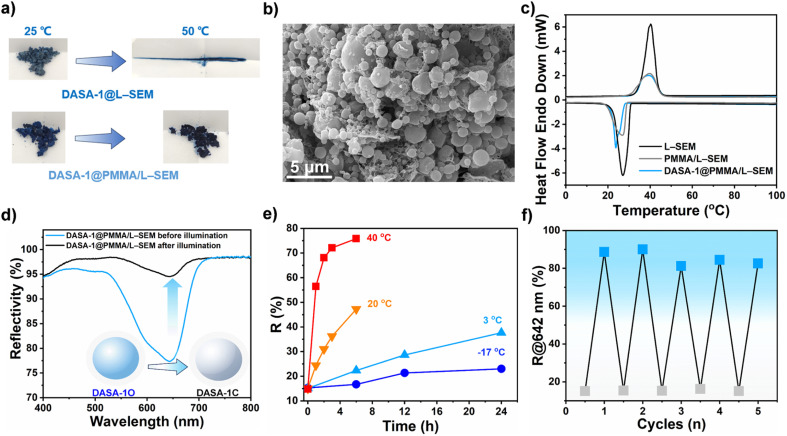
(a) Photos of DASA-1@L–SEM and DASA-1@PMMA/L–SEM capsules at various temperatures. (b) SEM image of DASA-1@PMMA/L–SEM capsules. (c) DSC curves of L–SEM, PMMA/L–SEM capsules, and DASA-1@PMMA/L–SEM capsules. (d) Reflectance spectra of DASA-1 capsules before and after 636 nm irradiation (41.3 mW cm^−2^, 5 min). (e) Time-dependent backward isomerization of DASA-1 within capsules kept in the dark after irradiation. (f) Multiple rounds of isomerization of DASA-1 within capsules upon heating (50 °C for 2 min) and 636 nm irradiation.

While the backward isomerization of DASA-1 within L–SEM proceeds slowly at 20 °C, fortuitously, an increased conversion rate was observed at moderately higher temperatures. However, due to the relatively low melting point of L–SEM (40.3 °C), a new challenge arises where heating promptly induces DASA-1@L–SEM to transition into a liquid state (Fig. 3a), rendering it unsuitable for rapid recovery at elevated temperature. Consequently, to enhance thermal stability, poly(methyl methacrylate) (PMMA) capsules encapsulating both DASA-1 and L–SEM, denoted as DASA-1@PMMA/L–SEM capsules, were fabricated utilizing an emulsion-solvent evaporation method (see ESI† for full experimental details). Additionally, PMMA/L–SEM capsules without DASA-1 were prepared for comparison to evaluate the effect of DASA-1 on capsule morphology and melting point.

As shown in Fig. 3a, the color of DASA-1@PMMA/L–SEM capsules closely resembles that of DASA-1@L–SEM, while in contrast, the PMMA/L–SEM capsules appear white (Fig. S6†). To confirm the successful fabrication of capsules, scanning electron microscopy (SEM) and differential scanning calorimetry (DSC) analyses were conducted. SEM images (Fig. 3b and S6†) reveal that the addition of DASA-1 does not affect capsule morphology, with both DASA-1@PMMA/L–SEM and PMMA/L–SEM capsules consisting of spherical particles in nano and micro dimensions. For DASA-1@PMMA/L–SEM capsules, nano-sized particles are concentrated around 380 nm, while micro-sized particles center at 1.8 μm.

Furthermore, DSC analysis (Fig. 3c) reveals that DASA-1@PMMA/L–SEM, PMMA/L–SEM, and pristine L–SEM exhibit similar endothermic and exothermic peaks, indicating that the latent heat of the capsules mainly derives from the L–SEM. Specifically, melting points were recorded at 40.3 °C for L–SEM, 39.7 °C for capsules without DASA-1, and 39.3 °C for capsules containing DASA-1, with corresponding latent heats of 205.3 J g^−1^, 115.4 J g^−1^, and 102.5 J g^−1^, respectively. These findings suggest that the addition of 3 wt% DASA-1 reduces the latent heat of fusion by 11.2%, likely due to DASA-1 interfering with the orderly packing of L–SEM molecules, leading to reduced crystallinity. Moreover, the solidification temperatures for L–SEM, capsules without DASA-1, and capsules with DASA-1 were recorded at 27.1 °C, 26.9 °C, and 23.7 °C, respectively, with corresponding latent heats of 186.8 J g^−1^, 91.4 J g^−1^, and 62.88 J g^−1^. Generally, the level of latent heat correlates with the concentration of fatty acids present. By comparing the latent heat values of the capsules with those of unencapsulated L–SEM during the melting process, encapsulation percentages of L–SEM were calculated as 49.9 wt% for capsules with DASA-1 and 56.2 wt% for those without, both lower than the expected 62.3 wt% based on formulation. This reduction is attributed to the influence of DASA-1 on the crystallization process, as confirmed by DSC and XRD analyses (Fig. 3c and 2e). Additionally, many capsules have dimensions within the nanometer range, where nano-effects may also contribute to this reduction.^48–50^

All the aforementioned data verify the successful fabrication of DASA-1@PMMA/L–SEM capsules. Thanks to the protective PMMA shell, as depicted in Fig. 3a, these capsules remained intact under conditions up to 50 °C (and even up to 100 °C), whereas DASA-1@L–SEM liquefied, thereby achieving the goal of enhancing thermal stability.

### 2.4. DASA-1 displays highly efficient and rapid reversible isomerization within capsules under mild conditions

During the fabrication of capsules, besides DASA-1 and L–SEM, water (serving as a dispersing medium), DCM (utilized for dissolving DASA-1, PMMA, and L–SEM), polyvinyl alcohol (acting as the suspending agent), and PMMA (as the capsule shell) were employed. Given the complexity of the system, will the encapsulation impact the isomerization of DASA-1? To address this question, the reflectance spectra of the capsules were collected. As shown in Fig. 3d, the maximum reflectance band remained at 642 nm, consistent with measurements taken before encapsulation. Remarkably, within just 5 min of irradiation, the reflectance decreased to 16% of its original value, representing a 13% greater reduction than observed before encapsulation. This amplified decrease may be attributed to the presence of ester bonds in PMMA, which potentially facilitate this greater reduction.^51^

Regarding the backward isomerization, the conversion rates were monitored at various temperatures. Fig. 3e demonstrates that the higher the temperature, the faster the backward isomerization rate. Initially, at 20 °C, around 6.2 h post-illumination, the DASA-1 capsules had regained 50% of their initial state, a recovery rate slightly longer than that of DASA-1@L–SEM (5.5 h). This finding suggests that DASA-1 exhibits similar isomerization behaviors within capsules as it does within L–SEM, indirectly indicating that the microenvironment surrounding DASA-1 remains largely unchanged. Moreover, at −17 °C, the isomerization rate of DASA-1 was the lowest, and molecular mobility appeared to be frozen. Notably, at 40 °C, the efficiency reaches over 70%. Particularly, at this temperature, the time required to recover half of the initial value is only about 18 min. Finally, at 50 °C (Fig. 3f), the backward isomerization reaches around 90%. Furthermore, after undergoing 5 heating and irradiation cycles, DASA-1 still exhibits high isomerization efficiency (>80%) within the capsules.

### 2.5. Sustainable DASA-1@PMMA/L–SEM capsules for the fabrication of rewritable papers

Building upon the highly efficient and rapid reversible isomerization of DASA-1 within PMMA/L–SEM capsules, we now extend this system towards a practical and environmentally sustainable application: the development of rewritable paper. As concerns about the environmental impact of traditional paper production grow, the need for alternatives that reduce waste and minimize the demand for recycling becomes increasingly urgent. Rewritable paper provides a compelling solution,^52–54^ as it can be used multiple times by erasing and re-writing, significantly cutting down on waste. By leveraging the photochromic properties of DASA-1 and the enhanced stability offered by PMMA/L–SEM encapsulation, we aim to fabricate both monochromatic and color-rich rewritable papers. These innovations not only support global sustainability efforts by reducing the consumption of disposable paper but also broadening the potential applications of DASA-1 in advanced materials science, paving the way for new environmentally friendly technologies.

#### 2.5.1. Monochromatic rewritable papers

**Fig. 4:**
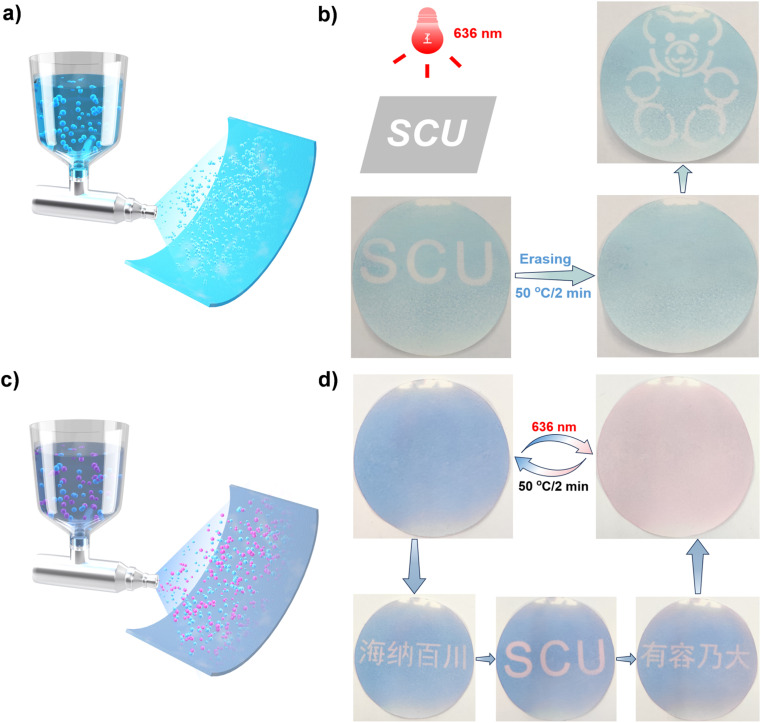
Schematic illustration of the fabrication of monochromatic rewritable papers using DASA-1 capsules (a) and color-rich rewritable papers using DASA-1 and DASA-2 capsules (c). (b) Photopatterning on the monochromatic paper in a positive manner, with photos captured after photopatterning and erasing. (d) Selective photopatterning on the color-rich paper in a positive manner, with photos captured after photopatterning and erasing.

Initially, we applied the aqueous solution of DASA-1 capsules onto a filter paper using the spray coating method (Fig. 4a), resulting in the fabrication of a monochromic rewritable paper. Fig. 4b demonstrates that, by employing specific photomasks, various patterns can be created on this paper. For example, under 636 nm illumination, the uppercase letters “SCU” (an abbreviation for Sichuan University) were formed on this surface. Subsequently, upon heating at 50 °C for 2 min, these letters nearly vanished. Again, by altering the photomask used we successfully achieved photoprinting of other patterns, such as a bear (top right corner of Fig. 4b).

#### 2.5.2. Color-rich rewritable papers

The utilization of monochromatic DASA-1 capsules limits the rewritable paper to switching only between sky blue and white, precluding the creation of richer color variations. To overcome this limitation, leveraging the ease of the color-tunable property of DASAs, we synthesized another DASA, termed DASA-2 (see ESI† for experimental details). To prevent potential adverse interactions between DASA-1 and DASA-2 within the same capsule that could negatively impact their respective isomerization, we exclusively prepared capsules containing only DASA-2. Specifically, using LA as the solid matrix and encapsulating it together with DASA-2 within PMMA capsules, DASA-2@PMMA/LA capsules were successfully prepared.

Fig. S7† displays the pink color of the DASA-2 capsules and the rose-red hue of the filter paper loaded with DASA-2 capsules, which are markedly distinct from those containing DASA-1. Subsequently, using the spray coating method with an aqueous solution containing both DASA-1 and DASA-2 capsules instead of DASA-1 capsules alone (Fig. 4c), a color-rich rewritable paper was prepared. As shown in Fig. 4d, the resulting paper appears ice blue, distinct from papers loaded with only one type of capsule. Since DASA-2 capsules are insensitive to 636 nm light, illuminating the paper with a 636 nm LED selectively activates only DASA-1 capsules. Heating then enables reversible color change of the paper between icy blue and pale pink (depicted in the upper half of Fig. 4d). Similar to before, through the use of photomasks and erasing at 50 °C (depicted in the lower half of Fig. 4d), the photoprinted patterns can be repeatedly photopatterned and erased. All the results demonstrate that DASA-1 capsules and the encapsulation technique described here can be used to fabricate rewritable papers, further expanding the scope of sustainable applications.

## 3. Discussion

The reversible isomerization of DASAs within solid matrices has garnered extensive attention. The core issue in this research area is whether DASAs can exhibit efficient isomerization behavior in solid matrices, similar to their performance in solution. While carefully engineered solid matrices are crucial, as emphasized here, it is undeniable that the molecular architecture of DASAs themselves also plays a significant role.

Although we have demonstrated that DASA-1 can undergo highly efficient isomerization within PMMA/L–SEM capsules, two questions arise: the first is whether all DASAs can achieve highly efficient isomerization within PMMA/L–SEM capsules under the same conditions. The second is what structural design considerations are necessary for DASAs to achieve high efficiency within PMMA/L–SEM capsules. Therefore, in the discussion section of this work, we attempt to address these two questions to inspire further research in this novel solid matrix direction.

**Fig. 5:**
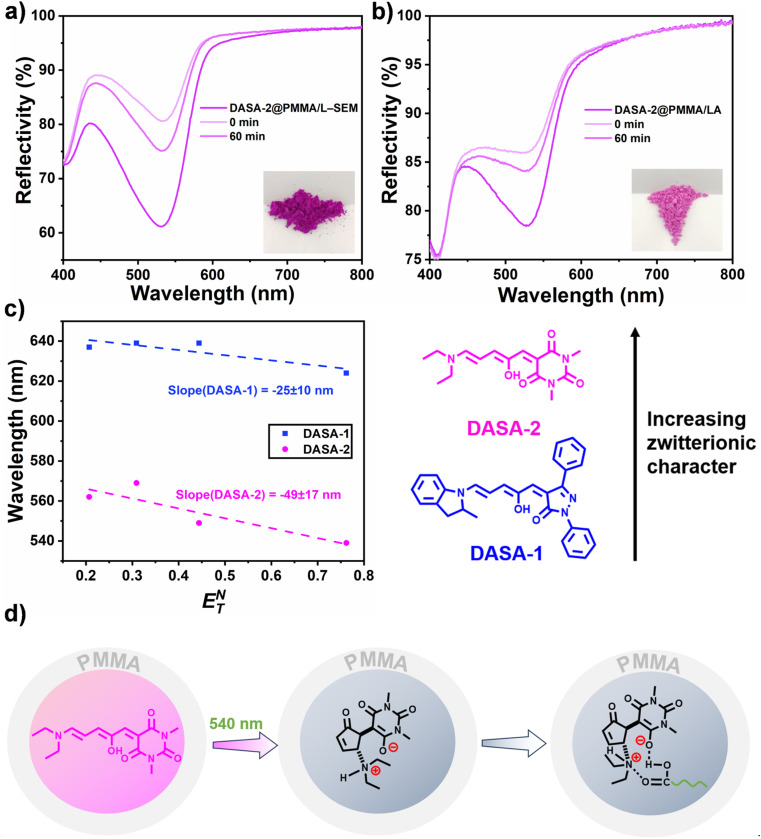
Thermal reversion of DASA-2 within PMMA/L–SEM (a) and PMMA/LA capsules (b) at 50 °C. The inset in (a) shows the sample of DASA-2@PMMA/L–SEM capsules before irradiation. (c) Solvatochromic shift analysis for DASA-1 and DASA-2 in solvents of varying polarities. (d) Presumed stabilization of the zwitterionic closed form of DASA-2 through its strong interactions with carboxylic acid groups in L–SEM.

Firstly, regarding the first question, the answer is no. Fig. 5a shows that even after 60 min at 50 °C, the backward isomerization efficiency of DASA-2 within PMMA/L–SEM capsules and PMMA/LA capsules only reaches 48% and 66%, respectively. This is significantly lower than that of the greater than 80% efficiency observed for DASA-1 in PMMA/L–SEM capsules.

Secondly, as for the second question, due to the limitations of molecular simulation conditions, we attempt to address it from the perspective of the differences in the zwitterionic nature of these two DASAs. According to Alaniz's work,^55^ the solvatochromic shift analysis was conducted. Fig. 5c exhibits that DASA-2 has a more negative solvatochromic slope than DASA-1 (−49 nm compared to −25 nm), suggesting that DASA-2 has a more pronounced zwitterionic nature than DASA-1. Due to the presence of carboxylic acid (–COOH) groups in fatty acids, the closed-ring isomer of DASA-2 may be more prone to interacting with them, resulting in higher backward conversion activation energy for DASA-2, and consequently limited backward isomerization efficiency (Fig. 5d). Overall, to achieve high efficiency in our system, the molecular design of DASAs must account for their zwitterionic nature, with a lower zwitterionic character being preferable.

Finally, it is important to highlight the unique aspects of our preparation method, which has successfully produced aqueous dispersions of DASAs. This capability is enabled by the inclusion of a small amount of hydrophilic PVA polymer (1 wt%) during the capsule preparation, allowing DASA capsules to disperse in water. Given the current challenges in controlling DASA isomerization behavior in aqueous solutions,^11,13^ this finding may offer a valuable avenue for addressing this issue. However, the uniformity of the capsule size is an issue that requires further resolution, and our laboratory is working on related improvements.

## 4. Conclusion

In summary, we have demonstrated that a DASA exhibits highly efficient and rapid reversible isomerization within sustainable nano/micro capsules, presenting a novel and environmentally friendly strategy for enhancing the photoswitching properties of DASAs in solid-state materials. Specifically, this work achieves an impressive forward isomerization efficiency of 84% at room temperature and a reverse isomerization of 90% at 50 °C within minutes. These results not only address the long-standing challenge of achieving efficient isomerization in high bio-content solid matrices but also emphasize the broader significance of sustainable materials for practical applications, such as color-rich rewritable paper. Furthermore, by deepening our understanding of DASAs' behavior within bio-based solid matrices—particularly through the innovative encapsulation with PMMA and eutectic mixtures—this research lays a solid foundation for future advancements in the field of smart materials. As a result, the encapsulation approach proposed here holds the potential to broaden the application of DASAs across various areas of chemical science, ultimately contributing to the development of eco-friendly technologies and global material sustainability.

## Data availability

The data supporting the findings of this work are available within the article and its ESI.† Raw data supporting the findings of this work are available from the corresponding author upon reasonable request.

## Author contributions

J. X. designed the experiments and supervised the project. J. X., BS. L. and H. M. performed the experiments and the data analysis. XN. F. and Q. Y. carried out the DSC testing and analyzed the data. YT. X. collected XRD curves and analyzed the data. J. X., Q. Y., HJ. F. and BS. L. co-wrote the manuscript. All the authors reviewed the manuscript.

## Conflicts of interest

There are no conflicts to declare.

## Supplementary Material

SC-OLF-D4SC04868G-s001
