# Supplementary material for: A DASA displaying highly efficient and rapid reversible isomerization within sustainable nano/micro capsules: one step closer to sustainability
Source: Chem Sci. 2024 Sep 25;15(41):17200–9. doi: 10.1039/d4sc04868g (PMC11441471; doi:10.1039/d4sc04868g)
Supplement: SC-015-D4SC04868G-s001 [file SC-015-D4SC04868G-s001.pdf]

Supplementary Information (SI) for Chemical Science.
This journal is © The Royal Society of Chemistry 2024

Supporting Information

A DASA Displaying Highly Efficient and Rapid Reversible Isomerization within Sustainable Nano/Micro Capsules: One Step Closer to Sustainability

Baoshuo Liu,[1] Xinnian Fan,[3,4] Hao Ma,[1] Yutong Xie,[1] Haojun Fan,[1] Qiang Yan,[2] and Jun Xiang[1,]*

[1]College of Biomass Science and Engineering, Sichuan University, Chengdu 610065, China

[2]State Key Laboratory of Molecular Engineering of Polymers, Department of Macromolecular Science, Fudan University, Shanghai 200433, China

[3]State Key Laboratory of Polymer Materials Engineering, College of Polymer Science and Engineering, Sichuan University, Chengdu 610065, China

[4]High-Tech Organic Fibers Key Laboratory of Sichuan Province, Chengdu 610041, China

*Emails: junxiang@scu.edu.cn;

1. Materials

Furfural (99%), 2-methylindoline (≥99%), benzoylacetic acid ethyl ester (BAEE, ≥95%), phenylhydrazine (PH, ≥98%), 1,3-dimethylbarbituric acid (DMBA, 99%), diethylamine (DA, ≥99%), 1,1,1,3,3,3-hexafluoro-2-propanol (HFIP, ≥99.5%), L-proline (99%), 2-methylindoline (98%), poly(methyl methacrylate) (PMMA, Mw = 350,000), polyvinyl alcohol (PVA, Mw = 31,000, 99% hydrolyzed), lauric acid (LA, ≥99%), stearic acid (SA, 99%) were supplied by Adamas-beta Reagent Co., Ltd (Shanghai, China). Dichloromethane (DCM, ≥98%) and acetic acid (≥99%) were supplied by Kelong Chemical Engineering Co. Ltd (Chengdu, China). Ultrapure water with a resistance of 18.2 MΩ·cm was obtained from a Millipore Simplicity 185 system.

2. Methods

^{1}H nuclear magnetic resonance (NMR) spectra were recorded at 25 ^{o}C using a 400 MHz Bruker AV III HD spectrometer. Tetramethylsilane was used as the internal standard. The molecular weight of DASA-1 was determined using a TSQ Quantum Ultra AM mass spectrometer (HermoFisherScienti, USA). Ultraviolet-visible (UV-Vis) measurements (including absorbance and reflectance) were carried out by a UV-Vis spectrophotometer (PE 1050, PerkinElmer, Inc., Boston, MA). Differential scanning calorimetry (DSC 200PC, Netzsch, Germany) was used to test the thermal properties of all samples. The sample (5~10 mg) was cooled to 0 ^{o}C and the heating to 100 ^{o}C at a rate of 10 °C min^{-1} in a nitrogen atmosphere with a purge flow of 50 mL min^{-1}. Meanwhile, the blank aluminum crucible was used as a reference. The red light used in the experiment was applied by a 636 nm-LED light source purchased from Shenzhen Nuosenda Electronics Co., Ltd. Scanning electron microscopy (SEM) images were collected from the Helios G4 UC scanning electron microscope (Thermo Fisher Scientific). Powder X-ray diffraction (XRD) measurements were performed on an Ultima IV diffractometer (Rigaku Corporation, Japan) equipped with Cu Kα radiation (λ = 1.5406 Å) in the range of 5–85° at room temperature.

3. Synthetic Procedures

3.1 Synthesis of DASA-1

The synthetic route of DASA-1 was depicted in **Scheme S1**. ^{1}H NMR spectra of products are presented in **Figure S1-S3**. The mass spectrum of DASA-1 is also provided in **Figure S3**.

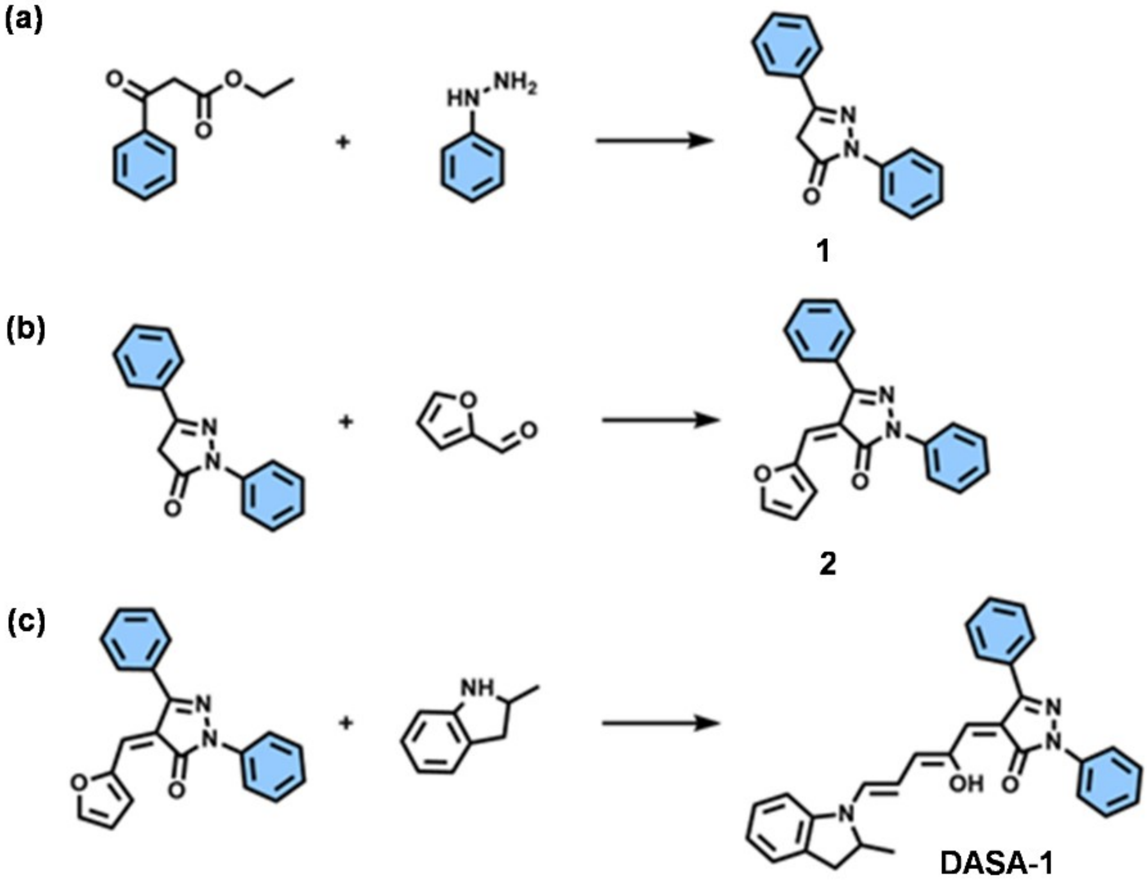

Scheme S1. The synthetic route of DASA-1.

Compound 1. A round-bottom flask equipped with a reflux condenser and a magnetic stir bar was charged with PH (4.35 mL) and BAEE (4.61 mL) dissolved in 0.4 mL of acetic acid. After stirring at 100 °C for 1 h under the protection of N_2, the reaction mixture was allowed to cool to 25 °C. The progress of the reaction was monitored using thin layer chromatography (n-heptane/ ethyl acetate, 3:2 v/v). The generated solid was filtrated and then recrystallization from ethanol afforded 2,5-diphenyl-2,4-dihydro-3H-pyrazol-3-one (**1**, 4.21 g, 74% yield).

Compound 2. A round-bottom flask equipped with a reflux condenser and a magnetic stir bar was

charged with compound **1** (1.0 g), L-proline (49 mg), and 2-furaldehyde (0.7 mL) dissolved in 30 mL of ethanol. After heating to reflux and stirring for 0.5 h under the protection of N_2, the reaction mixture was allowed to cool to -20 °C. The generated red precipitate was filtrated and washed with ice-cooled ethanol and then dried in a vacuum oven. The product was further purified by silica gel chromatography affording red-purple compound **2** ((*Z*)-4-(furan-2-ylmethylene)-2,5-diphenyl-2,4-dihydro-3H-pyrazol-3-one, 1.13 g, 85% yield).

DASA-1. Compound **2** (0.100 g), 2-methylindoline (0.100 g), and HFIP (0.2 mL) were dissolved in CH_2Cl_2 (0.8 mL). After reaction at 25 °C for 2 h, CH_2Cl_2 and HFIP were removed under reduced pressure. The remaining solid was triturated in diethyl ether. After filtration, DASA-1 was isolated as a dark blue solid (0.148 g, 51% yield).

3.2 Synthesis of DASA-2

DASA-2 was synthesized according to the method reported in the literature.[1] ^{1}H NMR spectra of products are presented in **Figure S8** and **S9**. Additionally, **Figure S10** shows the response of DASA-2 to green light (546 nm).

4. Preparation of DASA@L–SEM and DASA@LA

The preparation process of DASA@L–SEM was shown in **Scheme S2**. In a typical experiment, LA (400 mg) and SA (100 mg) were first dissolved in DCM (1.5 mL) in a vial. Then, the solution was added to a DCM solution (1 mL) containing 15 mg DASA-1 or DASA-2 under vigorous ultrasonic agitation with the help of an ultrasonicator (130 W, 20 kHz). The whole solution was stirred for 2 h before DCM was completed evaporation at normal pressure at room temperature. The remaining solid of DASA@L–SEM was ground into uniform powders and kept in the dark for subsequent characterization.

DASA@LA was prepared using the same procedure. In this case, solely LA (500 mg) was dissolved in DCM (1.5 mL) in a vial. The rest of the preparation was the same as described above.

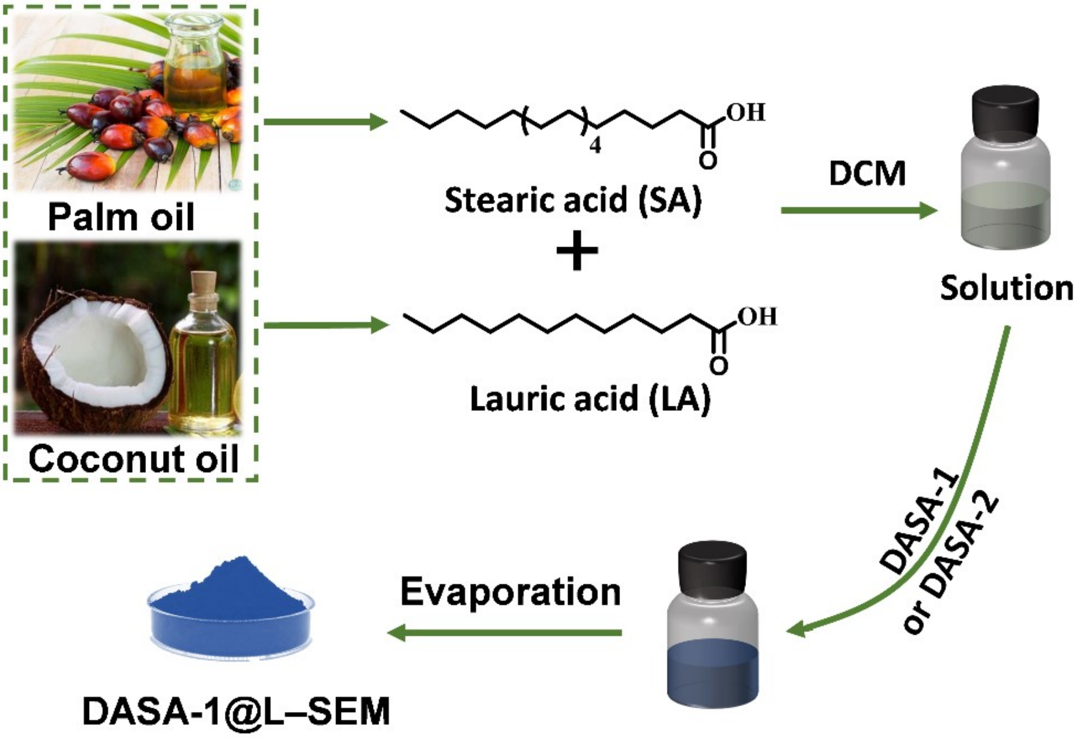

Scheme S2. Schematic diagram of the preparation of DASA@L–SEM powders.

5. Preparation of DASA@PMMA/L–SEM and DASA@PMMA/LA Capsules

In a typical experiment, the process began with the dissolution of 0.5 g of PMMA in 10.4 mL of DCM under magnetic stirring. Concurrently, 30 mg of DASA-1 or DASA-2 was dissolved in a mixture of solid fatty acids consisting of 0.72 g of LA and 0.18 g of SA. The dissolution of DASA-1 or DASA-2 triggered a noticeable color change. Once the complete dissolution of DASA-1 or DASA-2 was achieved, the LA/SA solution was introduced into the DCM solution. Subsequently, this organic mixture was added to a pre-prepared PVA water solution (15 mL, 1 wt.%). Homogenization of the resulting mixture at room temperature for 20 min at 11000 rpm facilitated the creation of an oil-in-water emulsion. The entire emulsion was then transferred to a round-bottom flask and subjected to heating at 44 °C under vacuum for 20 min, promoting the evaporation of DCM and the precipitation of PMMA around the SA/LA droplets. Following this, the resulting suspension was diluted with 30 mL of water and subjected to centrifugation at 12000 rpm for 10 min. This process was repeated three times, with capsules being redispersed in 20 mL of water each time. After freeze-drying the suspension in the dark, a fine solid powder of DASA-1@PMMA/L–SEM or DASA-2@PMMA/L–SEM was

obtained.

DASA@PMMA/LA capsules were prepared using the same procedure. In this case, 30 mg of DASA-1 or DASA-2 was dissolved in 0.903 g of LA by heating and stirring. The rest of the preparation was the same as described above.

6. Preparation of Rewritable Papers.

Taking the preparation of the monochromatic rewritable paper containing DASA-1@PMMA/L–SEM as an example. Initially, an aqueous dispersion of DASA-1@PMMA/L–SEM capsules was prepared at a concentration of 71.5 mg/mL. Aliquot 4 mL of the dispersion and use an airbrush (nozzle diameter 0.3 mm) to spray it evenly onto a 7 cm diameter filter paper. The sprayed filter paper was allowed to dry at 25 °C in the dark for 24 h, then it was placed in an oven at 50 °C for 20 min. After drying, the filter paper was stored at room temperature in the dark for 24 h, resulting in the monochromatic rewritable paper containing DASA-1@PMMA/L–SEM.

The monochromatic rewritable paper containing DASA-2@PMMA/LA was prepared using the same procedure. In this case, the concentration of DASA-2@PMMA/LA capsules was 71.1 mg/mL. The rest of the preparation followed the same procedure as described above.

The color-rich rewritable paper containing DASA-1@PMMA/L–SEM and DASA-2@PMMA/LA was prepared using the same procedure. In this case, 2 mL each of the aqueous dispersions of DASA-1@PMMA/L–SEM (71.5 mg/mL) and DASA-2@PMMA/LA (71.1 mg/mL) were used. The rest of the preparation followed the same procedure as described above.

Table S1. Comparison of the isomerization efficiency achieved in this work with efficiencies exceeding 80% reported in the literature.[a]

Refs	Solid matrix	Type	Bio-content[b]	Forward efficiency and time[c]	Backward efficiency and time[d]	Temp.[e]
This	Sustainable Nano/Micro Capsules	PMMA/L–SEM capsules	57%[f]	84%/300 s	90%/120 s	50 °C
[2]	Porous crystals	MOFs	n/a	100%/8 s	94%/76 s	60 °C
[3][g]	Sustainable amorphous polymers	PPU	52%	94%/20 s	92%/30 s	80 °C
[4]	Amorphous polymers	PPMA	n/a	88%/20 s	84%/30 s	70 °C
[5]	Amorphous polymers	PVP having PB	n/a	100%/56 s	97%/102 s	60 °C

a. Time to reach the corresponding efficiency ≤300 s.

b. Biocontent of the corresponding solid matrix.

c. Time to reach the corresponding backward efficiency.

d. Time to reach the corresponding forward efficiency.

e. Temperature for the backward isomerization.

f. See table S2.

g. Our previously published work.

Table S2. Bio-content of DASA-1@PMMA/L–SEM capsules.

Entry	Materials	Mass	Renewable or not	Ref.
1	PMMA	0.5 g	Not	
2	PVA	0.15 g	Not	
3	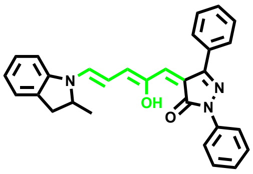 DASA-1	0.03 g	**Bio-content = 18%**	
4	Lauric Acid	0.72 g	Renewable	[6]
5	Stearic Acid	0.18 g	Renewable	[7]
6	DASA-1@PMMA/L–SEM capsules		**Bio-content = 57%**	

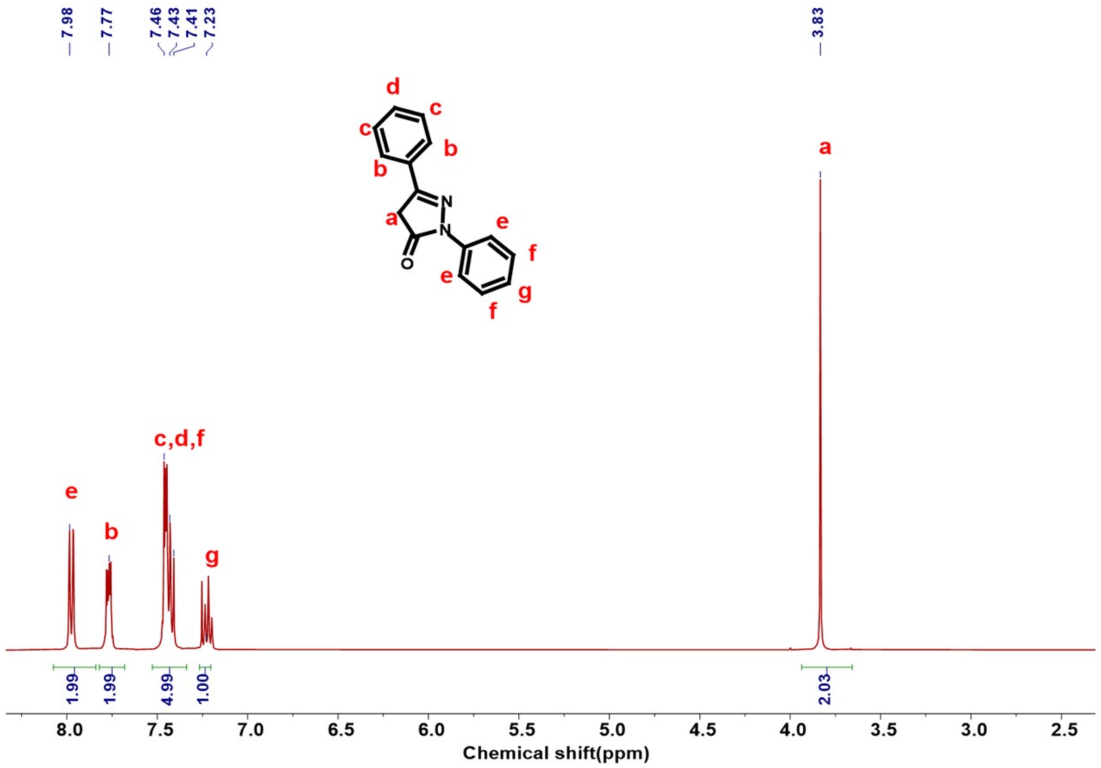

Figure S1. ^{1}H NMR spectrum of compound **1** in $CDCl_3$.

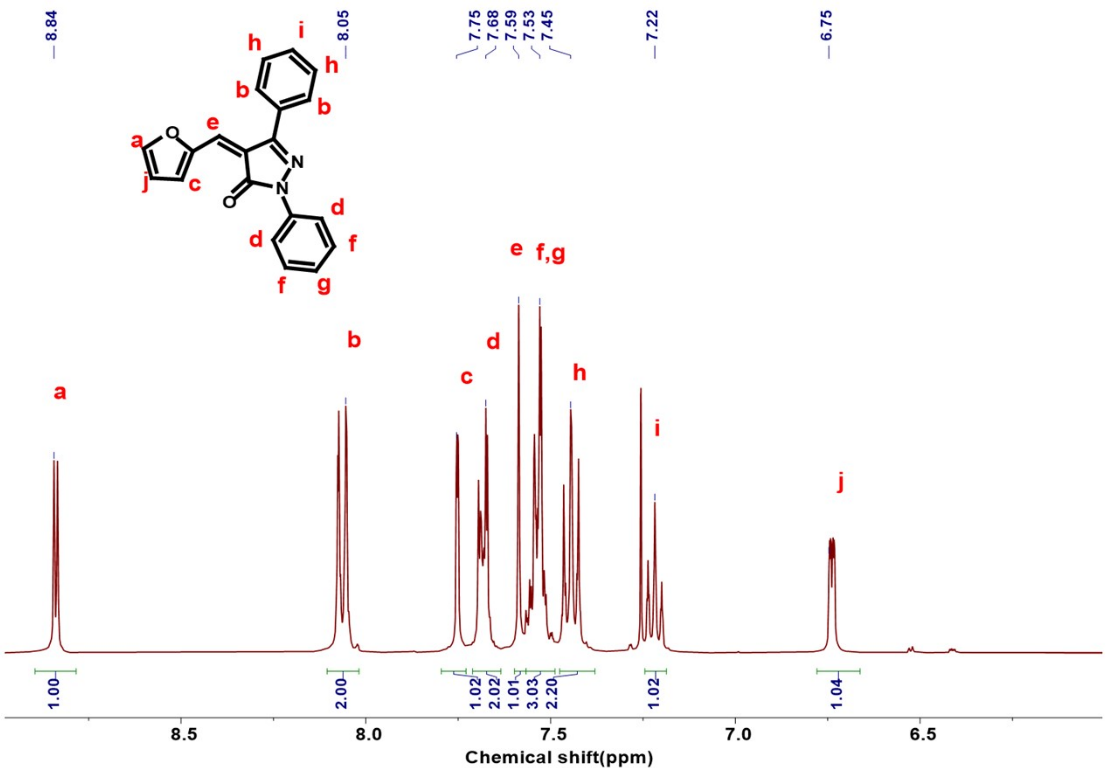

Figure S2. ^{1}H NMR spectrum of compound **2** in $CDCl_3$.

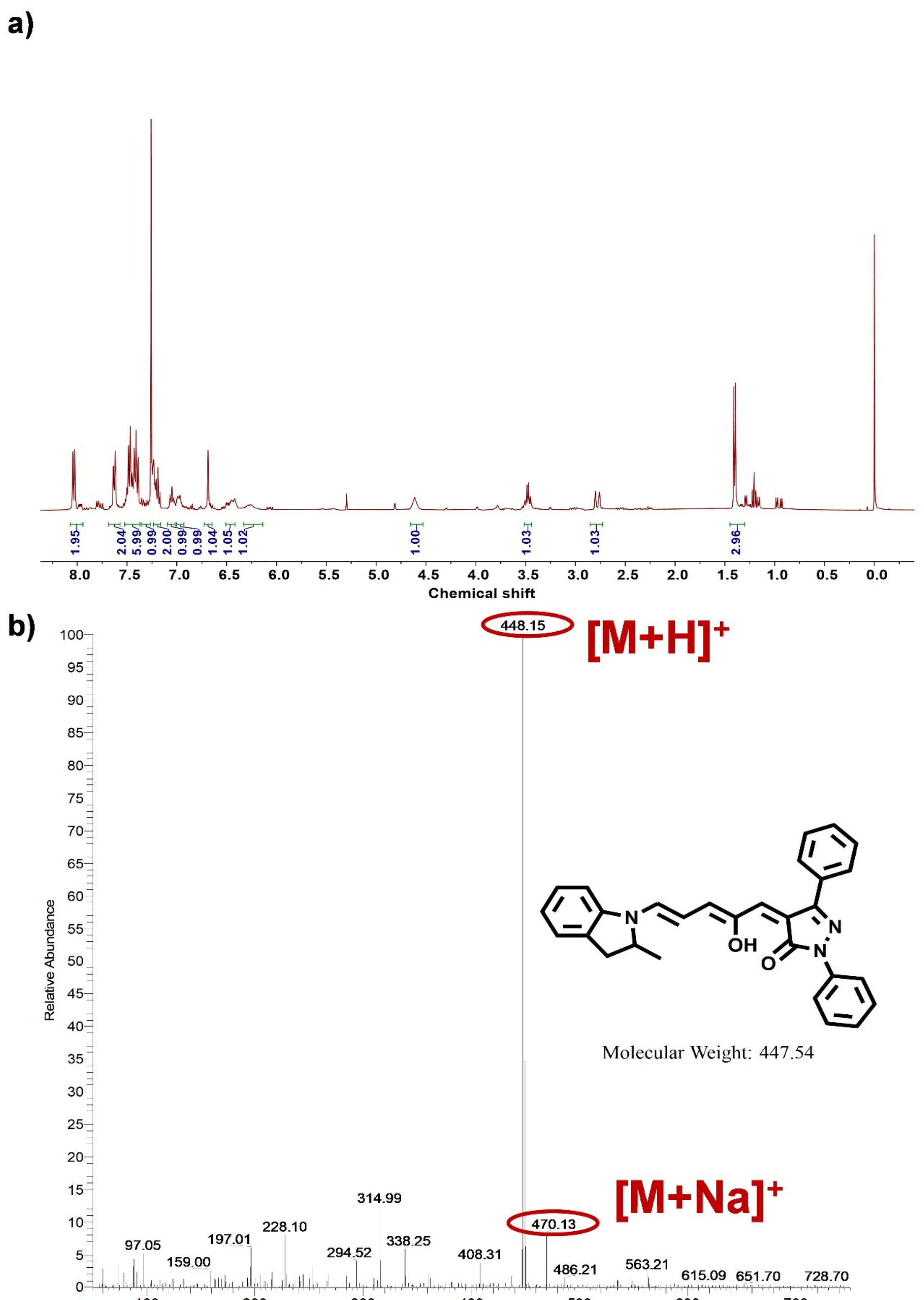

Figure S3. ^{1}H NMR spectrum (a) and mass spectrum (b) of DASA-1. Owing to the isomer equilibrium, it is difficult to confirm the successful synthesis of DASA-1 through the ^{1}H NMR spectrum; however, its mass spectrum confirms its high purity.

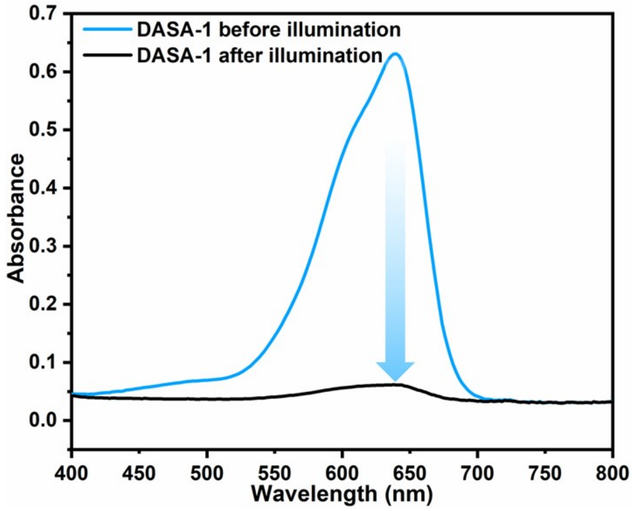

Figure S4. The absorption spectra of the DASA-1 solution before and after illumination at 636 nm (164 mW cm^{-2}, 5 s, 6.7 mmol L^{-1}, DCM, 25 ^{o}C).

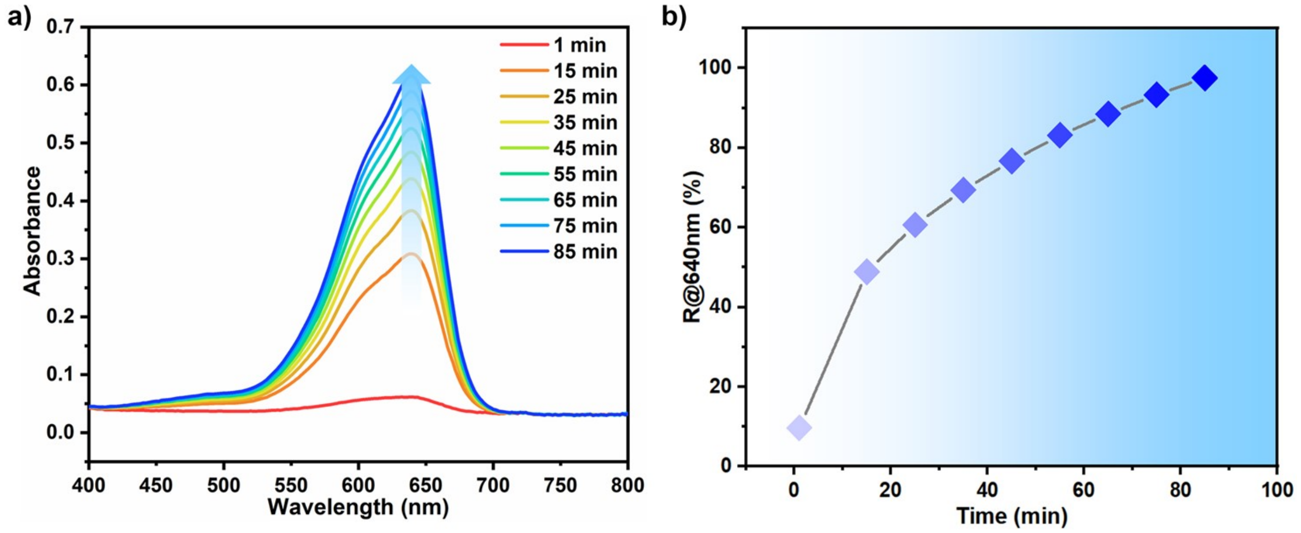

Figure S5. (a) Thermally reverts to the linear isomer of DASA-1 in the dark (25 ^{o}C, DCM) after activating with 636 nm irradiation. (b) The time kinetics of the backward *cyclic-linear* isomerization of DASA-1 in DCM at 25 ^{o}C.

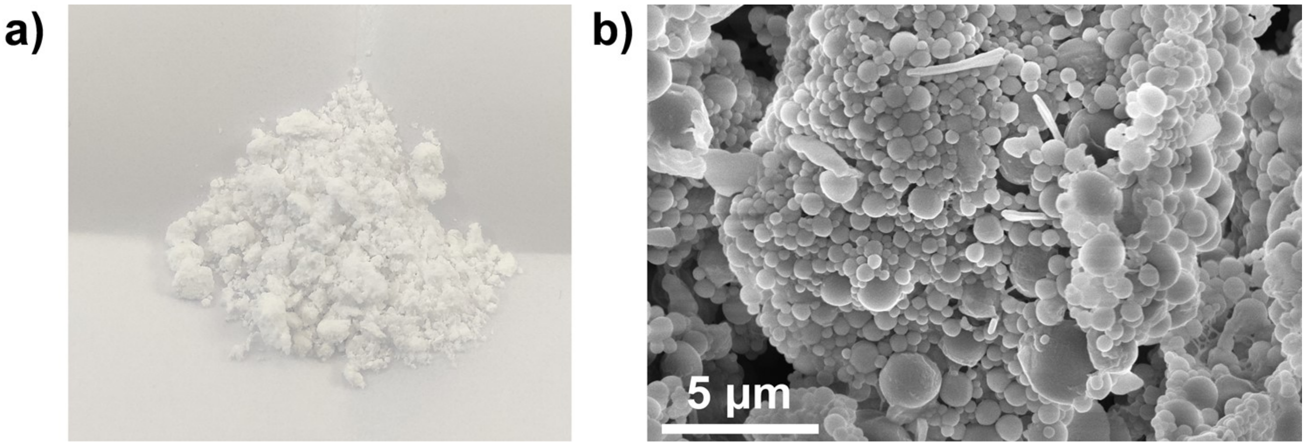

Figure S6. (a) Photo of PMMA/L–SEM capsules at room temperature. (b) SEM image of PMMA/L–SEM capsules.

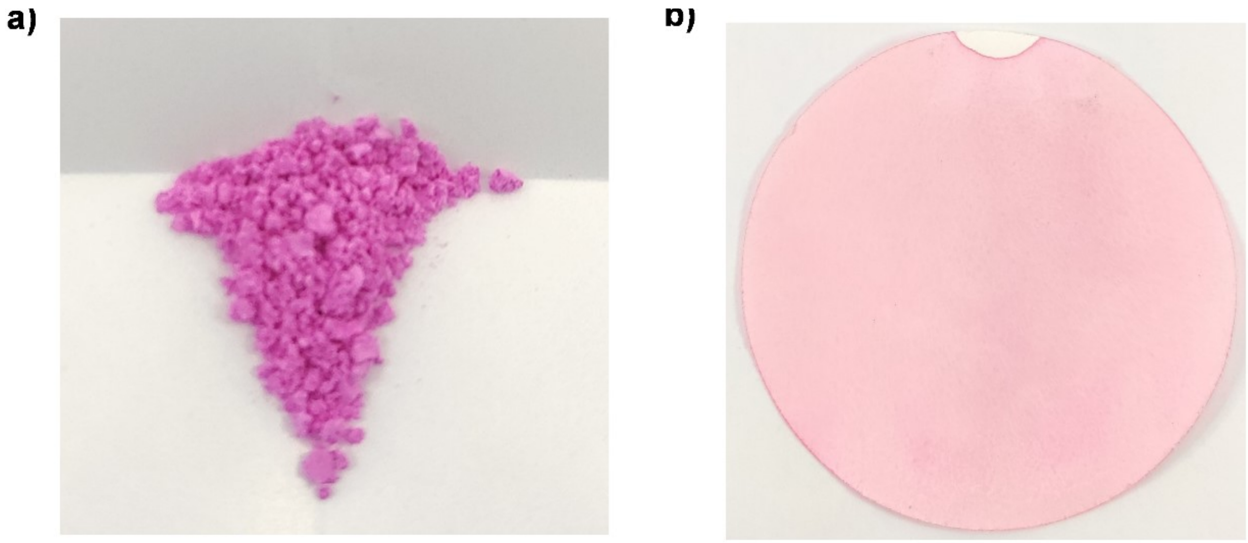

Figure S7. Photos of DASA-2@PMMA/LA capsules (a) and the filter paper loaded with DASA-2@PMMA/LA capsules (b) using the spray coating method.

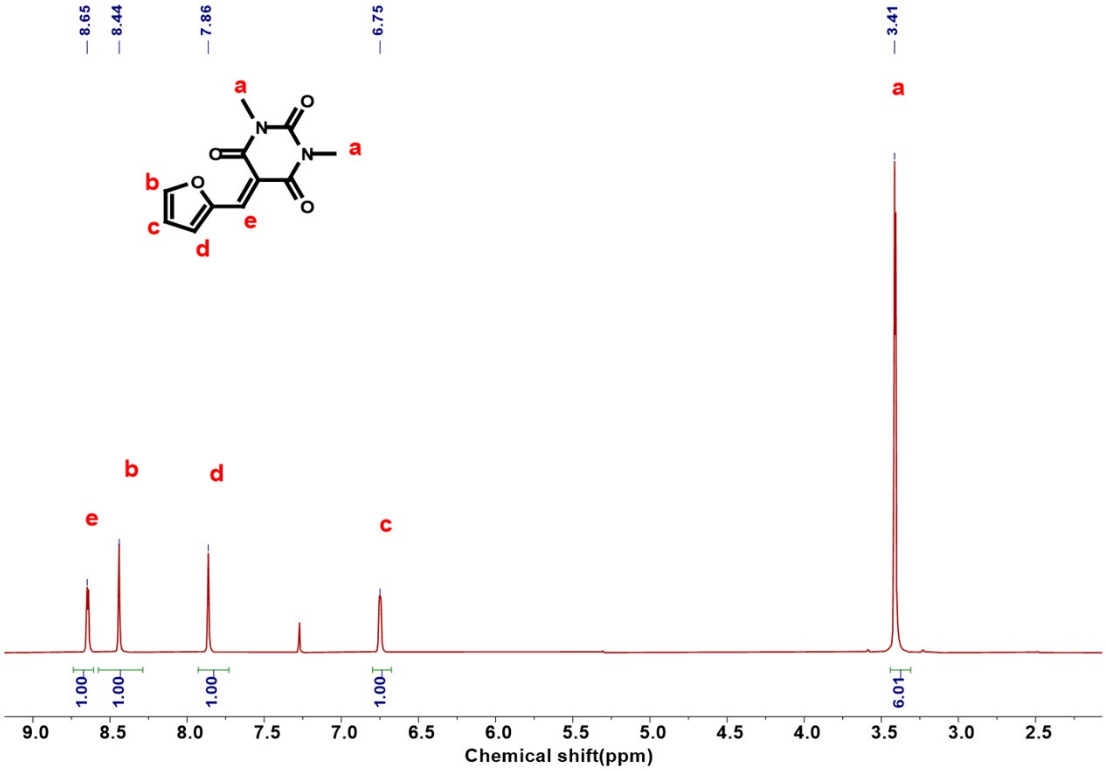

Figure S8. ^{1}H NMR spectrum of the acceptor of DASA-2 in $CDCl_3$.

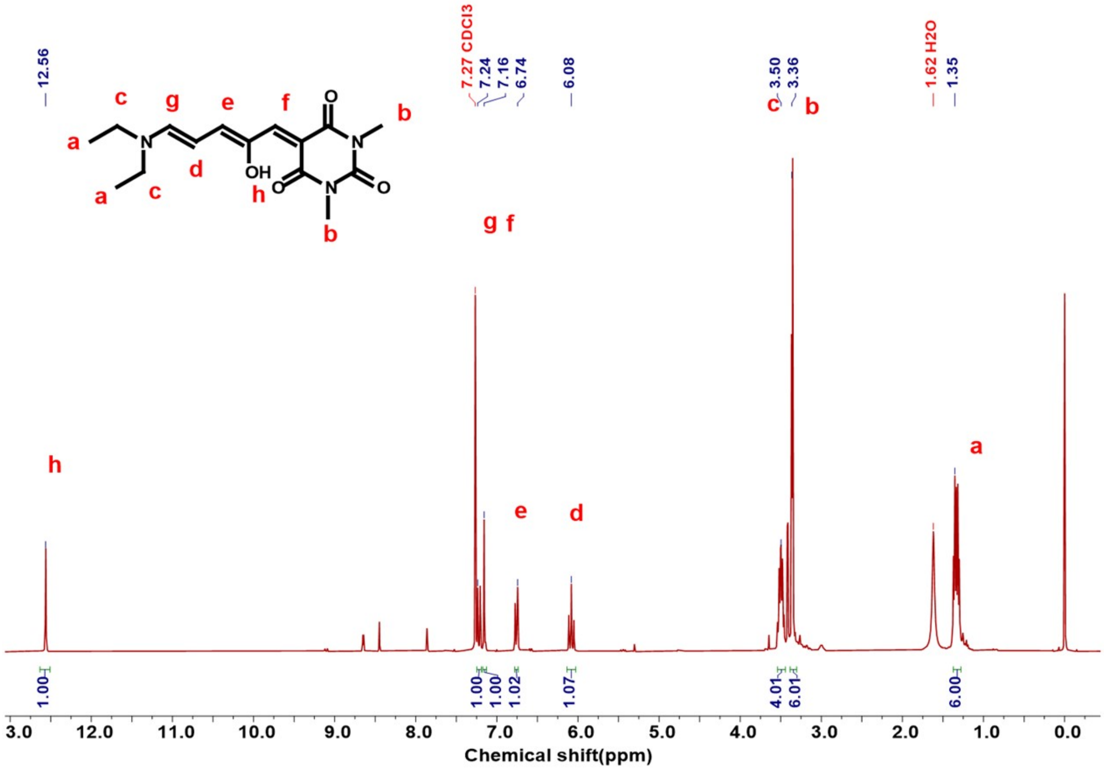

Figure S9. ^{1}H NMR spectrum of DASA-2 in $CDCl_3$. ^{1}H NMR data match the values reported by Park, et al.[8]

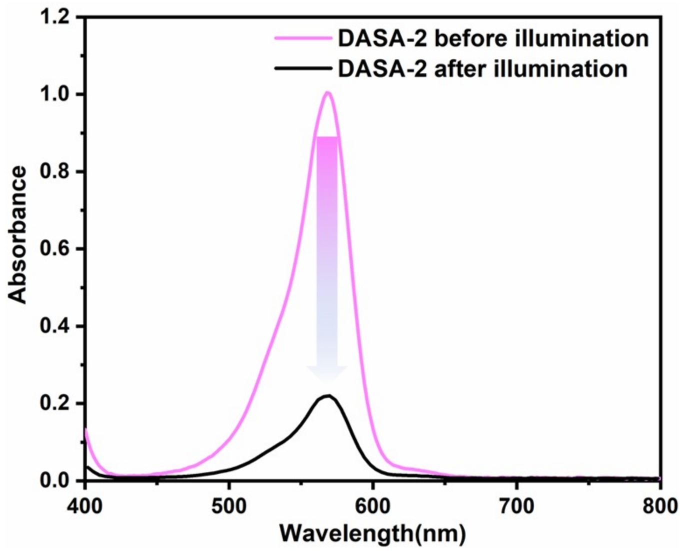

Figure S10. The absorption spectra of the DASA-2 solution before and after illumination at 546 nm (10 mW cm^{-2}, 5 s, paraxylene, 25 ^{o}C).

References

1. Helmy, S.; Oh, S.; Leibfarth, F. A.; Hawker, C. J.; de Alaniz, J. R., Design and Synthesis of Donor-Acceptor Stenhouse Adducts: A Visible Light Photoswitch Derived from Furfural. *Journal of Organic Chemistry* **2014,** *79* (23), 11316-11329.
2. Sun, F. X.; Xiong, X. Y.; Gao, A.; Duan, Y. L.; Mao, L. J.; Gu, L. H.; Wang, Z.; He, C.; Deng, X.; Zheng, Y. H.; Wang, D. S., Fast photochromism in solid: Microenvironment in metal-organic frameworks promotes the isomerization of donor-acceptor Stenhouse adducts. *Chemical Engineering Journal* **2022,** *427*, 132037.
3. Zhou, S.; Wang, L.; Wang, Y.; Ma, H.; Yan, B.; Guo, K.; Yan, Q.; Fan, H.; Xiang, J., Sustainable Indicators Based on Furfural-Derived Colorant-Doped Biobased Polyurethane to Improve Food Safety. *ACS Sustainable Chemistry & Engineering* **2022,** *10* (26), 8624-8630.
4. Sroda, M. M.; Lee, J.; Kwon, Y.; Stricker, F.; Park, M.; Valentine, M. T.; Read de Alaniz, J., Role of Material Composition in Photothermal Actuation of DASA-Based Polymers. *ACS Applied Polymer Materials* **2022,** *4* (1), 141-149.
5. Xiong, X.; Sun, F.; Gao, A.; Wang, Z.; Duan, Y.; Yao, Z.; He, C.; Han, R.; Deng, X.; Zheng, Y.; Wang, D., Ester matters? Promoting photoisomerization of donor–acceptor Stenhouse adducts in the solid state and "burn after reading" encryption. *Chemical Engineering Journal* **2022,** *450*, 138090.
6. Nitbani, F. O.; Tjitda, P. J. P.; Nitti, F.; Jumina, J.; Detha, A. I. R., Antimicrobial Properties of Lauric Acid and Monolaurin in Virgin Coconut Oil: A Review. *ChemBioEng Reviews* **2022,** *9* (5), 442-461.
7. Salas, J. J.; Martínez-Force, E.; Harwood, J. L.; Venegas-Calerón, M.; Aznar-Moreno, J. A.; Moreno-Pérez, A. J.; Ruíz-López, N.; Serrano-Vega, M. J.; Graham, I. A.; Mullen, R. T.; Garcés, R., Biochemistry of high stearic sunflower, a new source of saturated fats. *Progress in Lipid Research* **2014,** *55*, 30-42.
8. Choi, S. J.; Seo, E. J.; Bae, H. E.; Jung, H. C.; Lee, S. H.; Kim, J. C.; Jung, Y. J.; Park, J. S.; Jeong, J.-E.; Park, Y. I., Anti-counterfeiting fiber system with near-infrared wavelength selectivity based on photothermal and thermochromic dyes. *RSC Advances* **2024,** *14* (5), 3560-3566.
